# Study on the neuroprotective effects of Genistein on Alzheimer’s disease

**DOI:** 10.1002/brb3.2100

**Published:** 2021-03-11

**Authors:** Xiaoying Duan, Yanshuang Li, Fei Xu, Hong Ding

**Affiliations:** ^1^ Department of Acupuncture and Moxibustion the Second Hospital of Jilin University Changchun China; ^2^ Department of Traditional Chinese Medicine the Second Hospital of Jilin University Changchun China

**Keywords:** Alzheimer's disease, amyloid β‐protein, ApoE, cholinergic neurons, Estrogen, Genistein, neural regeneration, oxidative stress, SERMs, tau protein

## Abstract

Along with the aging of the world population, the incidence rate of Alzheimer's disease (AD) has been increasing. At present, AD has become one of the most serious problems faced by modern medicine. Studies have shown that estrogen has a positive effect on AD, but estrogen has the side effect of leading to tumors. Recent in vivo studies have shown that genistein, one of the selective estrogen receptor modulators (SERMs), can improve brain function through the blood–brain barrier (BBB), antagonize the toxicity of amyloid β‐protein (Aβ), that is, to inhibit neurotoxicity due to aggregation of beta amyloid protein, and have neuroprotective effects. In addition, the use of Gen can avoid the risk of endometrial cancer and breast cancer caused by estrogen therapy while exerting an estrogen‐like effect, which has some potential for the delay and treatment of AD.

## Graphical abstract

Recent studies have shown that genistein, one of the selective estrogen receptor modulators (SERMs), can improve brain function through the blood–brain barrier (BBB), antagonize the toxicity of amyloid β‐protein (Aβ), and have neuroprotective effects. In addition, the use of Gen can avoid the risk of endometrial cancer and breast cancer caused by estrogen therapy while exerting an estrogen‐like effect, which has some potential for the delay and treatment of AD.

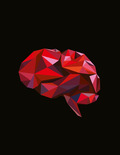

## 1 INTRODUCTION

Alzheimer's disease (AD), also known as senile dementia, is a chronic degenerative disease of the central nervous system. Its main clinical manifestation is progressive dementia, and it is characterized by mental symptoms, such as decreased cognitive function, memory and language dysfunction, and behavioral disorders of varying degrees. Epidemiological statistics show that the majority of patients with this disease are female, and the incidence rate in females over 65 years old is 15%, which is three times that of males of the same age.

Shi & Xu (2008) compared the activity of cytochrome c oxidase (COX) and the level of mitochondrial adenosine triphosphate (ATP) in the hippocampi of young and middle‐aged ovariectomized (OVX) rats. The results showed that the activity of COX and the level of mitochondrial ATP in the hippocampus of young OVX rats changed for a short time, while the activity of COX and the level of mitochondrial ATP in the hippocampus of middle‐aged OVX rats decreased for an extended period of time. This suggests that the onset of AD is related to an increase in age, especially to a decrease in ovarian function and a decrease in estrogen levels, and to a decrease in mitochondrial ATP and a decrease in mitochondrial ATP synthesis in the hippocampus (Shi et al., 2008).

At present, experimental studies and clinical observations have shown that estrogen has a protective effect on nerve cells. However, estrogen also has a proliferative and carcinogenic effect on non‐neuronal cells (mammary gland cells and endometrial cells; Beresford et al., 1997), greatly increasing the risks of using estrogen. Thus, phytoestrogen, which is similar in structure to estrogen and can combine with and activate the estrogen receptors of both nonhuman mammals and humans, is attracting much attention. Genistein (Gen) is the most active molecule in soybean isoflavones and can protect different kinds of cells from various toxic substances. It has been shown in in vivo studies that Gen can improve brain function through the blood–brain barrier (BBB), antagonize the toxicity of amyloid β‐protein, and have a neuroprotective effect. In addition, use of Gen, one of the selective estrogen receptor modulators (SERMs), can avoid the risk of endometrial cancer and breast cancer caused by estrogen therapy, while exerting an estrogen‐like effect, which has potential for delaying and treating AD. Therefore, Gen is an encouraging candidate to become an estrogen substitute for the prevention and treatment of AD. The effect of Gen on AD is summarized below.

### 1.1 The effect of Genistein on amyloid β‐protein

Amyloid β‐protein (Aβ) is hydrolyzed by β‐amyloid precursor protein (APP). In normal physiological conditions, APP secretes soluble Aβ, which can promote the growth of neurites, improve the survival rate of neurons, and protect the activity of neurons. However, in pathological conditions, APP secretes insoluble Aβ under the action of Beta‐site (βsite) APP cleaving enzyme 1 (BACE1). The insoluble Aβ has a strong neurotoxic effect after deposition and aggregation, leading to the apoptosis and dysfunction of AD neurons. It has been shown that Gen can reduce the production of Aβ by inhibiting BACE1 (Li et al., 2013; Youn et al., 2018). Kim et al. (2002) found that Gen can block the stimulating effect of platelet‐derived growth factor (PDGF) on APP and so reduce the secretion of APP. Hirohata et al. (2012) observed that among the fragments of Aβ—Aβ_1‐25_, Aβ_25‐35_, and Aβ_33‐42_—Aβ_25‐35_ showed the strongest degree of deposition. Genistein can directly bind the Aβ25‐35 fragment with the highest aggregation degree to prevent the formation of Aβ_25‐35_ aggregates and can also reduce the accumulation of Aβ_1‐25_ and Aβ_33‐42_ fragments. The data produced by Yu et al. (2010) showed that Gen can reduce the neurotoxicity induced by Aβ_42_ and Aβ accumulation by inhibiting Inhibition of kinesin AP180T (AP180) and Ras homolog family member A (RhoA).

### 1.2 Genistein's mediation of the anti‐inflammatory effect

Microglia is the first and most important line of defense in the central nervous system. The initial neurologic change in AD patients is the activation of microglia around the Aβ deposition site, which increases proinflammatory cytokine levels. In addition, nuclear factor‐B is an important transcription factor; inflammation caused by activation of the enhancement of κ‐light chain in nuclear factor‐activated B cells(NF‐κB) signal path is also the key to the onset of neurodegenerative diseases. Zhou et al. (2014) found that the expression of the Toll‐like receptor (TLR4) increases in microglia BV‐2 with Aβ_25‐35_ accumulation. After TLR4 is activated, it can activate the NF‐kB signal path by signal transduction. NF‐kB can cause inflammatory reactions by regulating the expression of several inflammatory mediators and cytokines. The results showed that the expression of TLR4 was inhibited by the microglia BV‐2 after pretreatment with 50 µM Gen for two hours, indicating that Gen can inhibit the activity of NF‐kB by blocking the NF‐kB signal path mediated by TLR4 to exert an anti‐inflammatory effect. This is consistent with the report from Seo et al. (2018) that Gen can block the binding of NF‐kB to target DNA that antagonizes the neuro‐inflammatory effect of AD. Jantaratnotai et al. (2013) reported that Gen reduced the production of nitric oxide(NO) in rat microglia induced by lipopolysaccharide (LPS) and lowered the level of interferon regulatory factor‐1 (IRF‐1) and STAT1, achieving anti‐inflammatory effects. The anti‐inflammatory effect of Gen is also reflected in the inhibition of inflammatory factor levels. When the microglia cell line HAPI was pretreated with Gen, it decreased the monocyte chemoattractant protein‐1, interleukin‐6 mRNA, and proinflammatory chemokines produced by the HAPI, stimulated by LPS. Valles et al. (2010) also demonstrated that levels of interleukin‐1β (IL‐1β) and tumor necrosis factor‐α (TNF‐α) were also controlled after astrocytes pretreated by Gen were induced by Aβ.

### 1.3 The effect of Genistein on cholinergic neurons

Dysfunction of the central cholinergic nervous system (CNS) and lack of neurotransmitter acetylcholine (ACH) are the key mechanisms of cognitive decline in AD patients (Pedersen et al., 1996; Rajput & Sarkar, 2017). It has been shown that Gen can improve cognitive disorder in diabetic mice by inhibiting the activity of acetylcholinesterase (AChE) because Amyloid β‐protein reduces acetylcholine synthesis in cell lines of basal forebrain cholinergic neurons (Pedersen et al., 1996). Amyloid β‐protein can induce Ca^2+^ to enter cholinergic neurons in the basal forebrain cortex—a region closely related to learning, memory, and cognition—through voltage‐gated channels, eventually leading to the death of cholinergic neurons. (Weiss et al., 1994). In the presence of the tyrosine kinase inhibitor Gen, the extracellular current of the cholinergic neurons in the basal forebrain nucleus diagonal belt (DBB) decreases and the influx of Ca^2+^ decreases. Thus, the protective effect of Gen on cholinergic neurons is achieved through the ion channels (Jhamandas et al., 2001). J H Jhamandas et al. used nuclear diagonal band (DBB) in the basal forebrain cholinergic neurons as the research object and observed that Gen on the neurotoxicity of DBB induced by Aβ_25‐35_ and Aβ_1‐40_, the results found that Genistein can inhibit tyrosine kinase inhibitors induced the Ca^2+^ K^+^ channels open, and inhibit the acetylcholine receptor nicotinic acetylcholine receptors (nAChRs) induced Ca^2+^ release, nAChRs is ionic receptors, visible, The protective effect of Gen on cholinergic neurons is realized through the ion channel pathway of neurons (Jhamandas et al., 2001). Orhan et al. have also reported that quercetin, Gen, and various flavonoids have different degrees of inhibitory effect on AChE and butyrylcholinesterase (BChE), reducing the damage caused by ACH (Orhan et al., 2007). Guo J et al. observed that Gen effectively inhibit the reduction of 7nAChR in hippocampal neurons induced by Aβ_25‐35_ (Guo J, 2004). Some Gen derivatives also show a good inhibitory effect on AChE (Qiang et al., 2014; Shi et al., 2012), thus improving cognitive disorder and dysmnesia in mice.

### 1.4 Genistein's inhibition of phosphorylation of tau protein

The abnormal hyperphosphorylation of microtubule‐associated protein tau leads to neuronal fiber entanglement (NFT), which is one of the pathological features of AD. The brain of an AD patient is extensively destroyed by the microtubule structure of the neurons. The normal axonal transport is damaged, the function of the synaptic‐loss neurons is damaged, and neurodegeneration occurs. Tau protein can be phosphorylated by CAMK4, and its hyperphosphorylation leads to the formation of NFTs (Miyano et al., 1992). Ye et al. found that both CAMK4 and tau protein increased significantly in the hippocampi of AD model rats (Ye et al., 2017). Before construction of the AD rat model, the level of CAMK4 and tau protein decreased after seven days of pretreatment (1 ml/100 g bodyweight) with Gen. Therefore, Gen may decrease the hyperphosphorylation of tau protein by regulating CAMK4 in AD model rats.

Calmodulin‐dependent protein kinase II (CaMKII) also participates in phosphorylation of tau protein, increasing the expression of CaMKII in human neuroblastoma cells **(**SH‐SY5Y) induced by Aβ_25‐35_. Xi et al. (2016) pretreated SH‐SY5Y cells with Gen (sh‐SY5Y cells were pretreated with Gen of 50 µM for 2 hr, and then, Aβ_25–35_ was added), CaMKII and pCREB levels were significantly down‐regulated. The results showed that preadded Gen group could significantly down‐regulated CaMKII and pCREB levels in SH‐SY5Y, suggesting that Gen may reduce the phosphorylation of tau through the CaMKII–CREB signal path and resist synaptic toxicity (Xi et al., 2016). Genistein can also reduce tau phosphorylation and tau phosphorylation‐related kinase in WT and Apolipoprotein E(ApoE) (−/−), as well as the level of glycogen synthase kinase 3β (GSK‐3β) (Park et al., 2016). Park et al. observed that Gen protected SH‐SY5Y cells from homocysteine‐mediated DNA damage, endoplasmic reticulum stress(ER stress), apoptosis, and tau hyperphosphorylation (Park et al., 2010).

### 1.5 Genistein's regulation of the expression of Apolipoprotein E

Apolipoprotein E (ApoE) participates in many aspects of the pathogenesis of AD. It has an endocytosis effect on soluble starch precursor protein APP and has a strong affinity with Aβ. There are a large number of ApoE receptors in the brain. The receptors combine with ApoE and ingest Aβ, thus reducing the extracellular level of Aβ and reducing nerve injury (Pappolla et al., 1997). Apolipoprotein E promotes axonal growth. The basal cholinergic neurons in the forebrain, in particular, are more dependent on ApoE because the basal cholinergic synapses in the forebrain significantly reduce while the other cerebral cholinergic neurons remain unchanged in ApoE‐deficient mice (Bonet‐Costa et al., 2016; Kleifeld et al., 1998). Apolipoprotein E in the brain participates in the normal metabolism of lipids, which may lead to transport deficiency of cholesterol and phospholipid and lead to insufficient acetylcholine synthesis. In the brain, ApoE is mainly synthesized by astrocytes and is activated by RXR/PPARγ. Genistein can increase (up‐regulate) PPARγ induced by Aβ to increase the release of ApoE and decrease the deposition of Aβ.

### 1.6 The effect of Genistein on mitochondria and oxidative stress

The destruction of mitochondrial structures increases the production of oxygen free radicals. The active oxygen free radicals produced by mitochondrial destruction also act as a signal to initiate the mitochondrial apoptosis pathway and attack the mitochondrial membrane, which causes a change in the mitochondrial matrix volume, a decrease of ATP production, and eventually cell death (Wei et al., 2010; Zhang et al., 2007).

Shi et al. observed that Gen could restore the synthetic ratio of mitochondrial ATP in the hippocampi of OVX rats (Shi et al., 2008). Genistein also inhibited neuronal oxidant hydrogen peroxide levels induced by Aβ and decreased the release of cytochrome C, preventing mitochondrial toxicity induced by Aβ (Viña, Lloret, & Vallés, 2007; Viña, Lloret, & Vallés et al., 2007). 8‐Hydroxy‐deoxyguanosine (8‐OHdG) is currently the most common and reliable biomarker of mitochondrial DNA (mtDNA) damage (Fenga et al., 2017).

Ma et al. studied the protective effect of Gen on mtDNA damage induced by Aβ_25‐35_ in glioma cell C6 (Ma et al., 2013). The Aβ_25‐35_ increased the level of 8‐OHdG in C6 cells, the mRNA and protein expression of 8‐oxyguanine DNA glucoamylase (OGG1) in C6 mitochondria, and manganese superoxide dismutase (MnSOD) in mitochondria. The levels of 8‐OHdG, mRNA, and the protein expression of OGG1 and MnSOD in C6 cells pretreated with Gen showed a decreasing trend.

Mitochondria may release proapoptotic gene cytochrome C, which induces activation of caspase and eventually induces apoptosis (Su Sin et al., 1999). Wang et al. established an AD rat model and (Wang et al., 2016) found that Gen can reduce hippocampal nerve injury by decreasing the expression levels of the cytochrome C, Bax, and caspase‐3 , suggesting that Gen may protect the hippocampal neurons of AD rats through the mitochondrial apoptosis pathway.

Manganese superoxide dismutase can eliminate superoxide free radicals and play a key defensive role in mitochondrial dysfunction. The absence of MnSOD also increases the formation of plaques and NFTs in the AD brain (Esposito et al., 2006; Melov et al., 2007). The decrease of impaired mitochondrial ATP in the AD brain results in a decrease in Na, K‐ATPase, and neurological function (Hattori et al., 1998; Kairane et al., 2002). Manganese superoxide dismutase is an important prerequisite for maintaining the normal function of Na and K‐ATPase. Kairane et al. compared the effects of three antioxidants on the activity of MnSOD, Na, and K‐ATPase in the AD brain (Kairane et al., 2014). The results showed that Gen can directly eliminate free radicals and increase the expression of MnSOD. Genistein also increased the activity of Na and K‐ATPase in the cerebral cortex of the AD brain, and there was a correlation between the present concentration and time.

Genistein has strong antioxidant properties, which can eliminate reactive oxygen species (ROS) and free radicals (Vallés et al., 2008) and enhance antioxidant enzyme activity (Cai & Wei, 1996). Genistein also shows a good antioxidative effect in the prevention and treatment of AD (Polkowski & Mazurek, 2000). Genistein can inhibit the pro‐oxygenic agent action of 24‐hydroxylatedcholesterol (24‐hydroxycholesterol), prevent neuronal necrosis and apoptosis induced by Aβ (Gamba et al., 2011), inhibit overproduction of ROS induced by Aβ_25‐35_ (Andersen et al., 2003) in rat brain synapses, and inhibit the influx of intracellular Ca^2+^ and the production of free radicals (Zeng et al., 2004). In addition, Gen also inhibited the oxidation of hemoglobin and myoglobin induced by hydrogen peroxide in AD rats (Boadi & Johnson, 2014; Occhiuto et al., 2009), indicating that Gen can play a role in neuroprotection through antioxidant activity.

**FIGURE 1:**
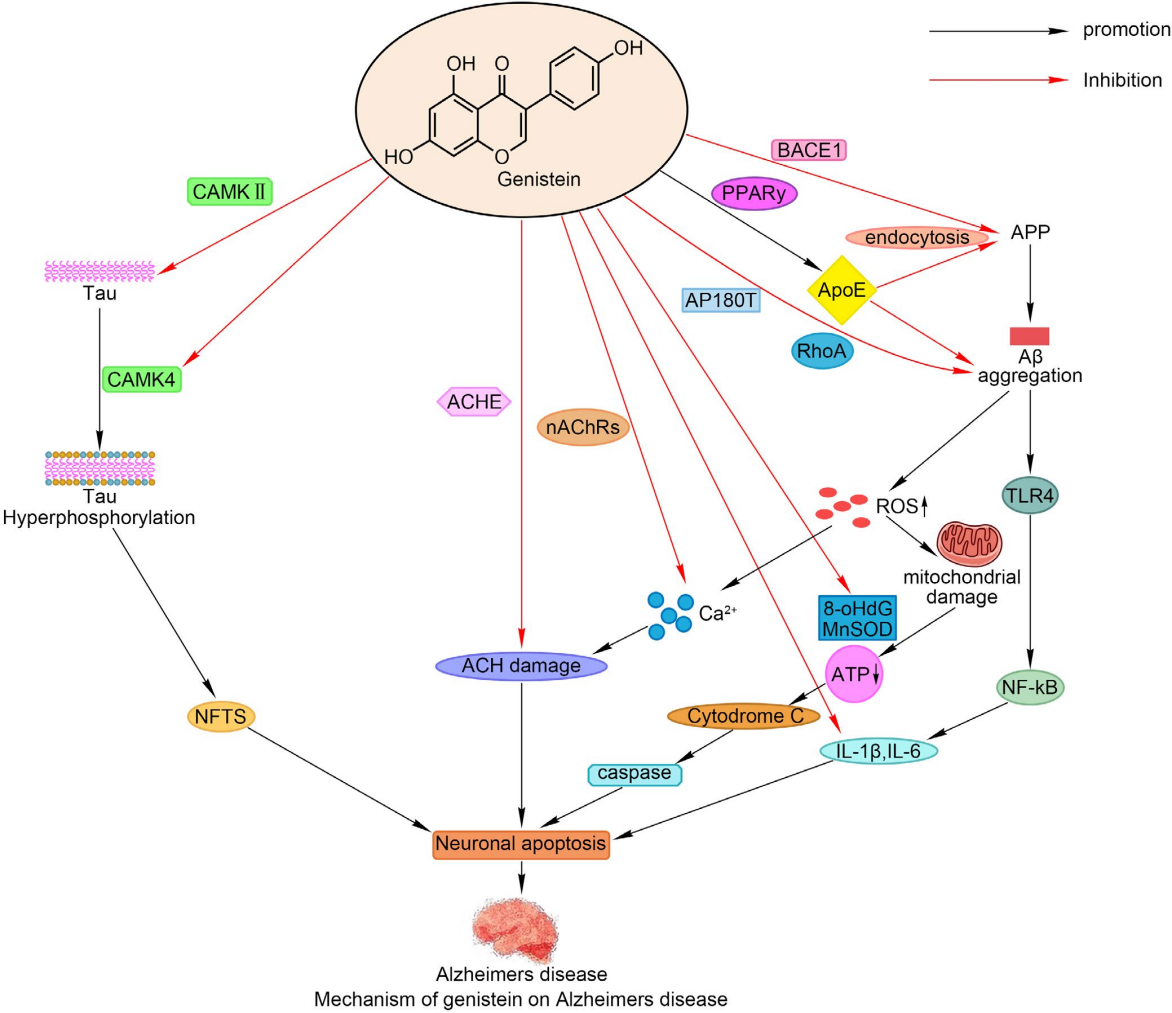
Gen's proposed mechanisms of action

Alzheimer's disease is a common and destructive neurodegenerative disease. In China, the number of AD patients is the highest in the world. The prevention and treatment of AD has become a topic of worldwide concern. Genistein is a natural isoflavone compound found in plants, which not only selectively combines with estrogen receptor ER‐β and exerts an estrogen‐like effect but also has unique antitumor effects (Radzikowski et al., 2004; Yu et al., 2005). Gen has strong antioxidant properties, which can eliminate reactive oxygen species (ROS) and free radicals (Vallés et al., 2008), improve the activity of antioxidant enzymes (Cai & Wei, 1996), and also play a good antioxidant stress role in the prevention and treatment of AD (Polkowski & Mazurek, 2000). In this study, through a literature review, we have found that Gen can delay and improve the harmful effects of AD through many mechanisms and targets both in in vivo and on the intracellular and extracellular molecular level (Figure 1), suggesting that Gen is a good alternative to substitute for estrogen in the treatment of AD.

## CONFLICT OF INTEREST

All of the authors confirm they have no conflict of interest to declare.

## AUTHOR CONTRIBUTION

Xiaoying Duan conceived the idea and conceptualized the study. Yanshuang Li collected the data. Fei Xu and Hong Ding analyzed the data. Xiaoying Duan and Hong Ding drafted the manuscript, and then, Xiaoying Duan and Hong Ding reviewed the manuscript. All authors read and approved the final draft.

### PEER REVIEW

The peer review history for this article is available at https://publons.com/publon/10.1002/brb3.2100.

[Correction added on March 20, 2021, after first online publication: Peer review history statement has been added.]
